# “Don’t talk to me like I am an illness”: exploring patients’ needs using the communication passport in an eating disorder service

**DOI:** 10.1007/s40211-024-00501-7

**Published:** 2024-07-12

**Authors:** Zhuo Li, Dimitri Chubinidze, Philippa Croft, Jessica Webb, Amanda Sarpong, Elisa Zesch, Kate Tchanturia

**Affiliations:** 1 Department of Psychological Medicine, Institute of Psychiatry, Psychology and Neuroscience, King’s College London, London, UK; 2 National Eating Disorder Service, South London and Maudsley NHS Foundation Trust, London, UK; 3 Department of Psychology, Ilia State University, Tbilisi, Georgia

**Keywords:** Eating disorder, Autism, Communication, Support, Patient needs, Essstörung, Autismus, Kommunikation, Unterstützung, Patientenbedürfnisse

## Abstract

**Purpose:**

Social challenges are common in patients with eating disorders (ED). The presence of autistic characteristics often exacerbates social difficulties within this group, potentially affecting treatment outcomes. This study investigates the communication preferences, challenges, dislikes, and support needs of patients with ED, both with and without autistic traits, using a communication passport in a national inpatient ED service.

**Methods:**

An explorative qualitative analysis of 38 completed communication passports was conducted to investigate patients’ communication preferences, sensory needs, struggles and dislikes, and areas of support required, paying particular attention to the distinct needs of patients with high levels of autistic traits.

**Results:**

The communication passport provided valuable insights into patients’ communication preferences, sensory sensitivities, challenges, and support needed. Patients also used the passports to share information about their strengths, personal identity, and life beyond the hospital.

**Conclusion:**

The communication passport fosters a deeper understanding of patients’ needs and may support clinicians in care planning and communication strategies tailored to each patient’s needs. Regular evaluation and updates are warranted to ensure its usability and accessibility by the wider care team.

## Abstract (de)

**Ziel:**

Bei Patienten mit Essstörungen sind soziale Herausforderungen häufig. Das Vorliegen autistischer Merkmale verstärkt oft soziale Schwierigkeiten innerhalb dieser Gruppe, was sich möglicherweise auf die Therapieergebnisse auswirkt. In der vorliegenden Studie wurden die Kommunikationsvorlieben, Probleme, Abneigungen und der Unterstützungsbedarf von Patienten mit Essstörungen, sowohl mit als auch ohne autistische Züge, anhand eines Kommunikationspasses in einer nationalen stationären Einrichtung für Patienten mit Essstörungen untersucht.

**Methoden:**

Es wurde eine explorative qualitative Analyse von 38 ausgefüllten Kommunikationspässen durchgeführt, um die Kommunikationsvorlieben, sensorischen Bedürfnisse, Probleme und Abneigungen sowie Bereiche mit Unterstützungsbedarf der Patienten zu untersuchen, wobei insbesondere Aufmerksamkeit auf die speziellen Bedürfnisse von Patienten mit stark ausgeprägten autistischen Zügen gelegt wurde.

**Ergebnisse:**

Durch den Kommunikationspass boten sich wertvolle Einblicke in die Kommunikationsvorlieben, sensorischen Empfindlichkeiten, Probleme und den Unterstützungsbedarf der Patienten. Dabei nutzten die Patienten die Pässe auch, um Informationen über ihre Stärken, persönliche Identität und das Leben außerhalb des Krankenhauses mitzuteilen.

**Schlussfolgerung:**

Der Kommunikationspass fördert ein tieferes Verständnis der Bedürfnisse von Patienten und hilft den klinisch Tätigen möglicherweise bei der individuell auf die Bedürfnisse des einzelnen Patienten abgestimmten Behandlungsplanung und Kommunikationsstrategie. Um seine Verwendbarkeit und Zugänglichkeit durch das erweiterte Behandlungsteam zu sichern, müssen regelmäßig Evaluationen und Aktualisierungen durchgeführt werden.

## Introduction

Anorexia nervosa (AN) is a complex illness characterised by profound socio-emotional and interpersonal difficulties [34]. Individuals with AN often exhibit attentional bias towards negative social signals [6, 11], struggle to accurately interpret the intentions and emotions of others [26], and encounter difficulties in expressing and articulating their own emotions [36]. Additionally, they may face challenges in cognitive perspective-taking or theory of mind [32]. Research suggests that social phobia and anxiety are common precursors to the development of eating disorders (EDs) and can increase vulnerability [8]. These social challenges persist into the acute phase of the illness [38] and may endure throughout the recovery process [25].

Autism, a neurodevelopmental condition characterised by challenges in social communication, sensory sensitivities, and repetitive behaviours and interests [3], often coexists with AN [37]. Autistic people often struggle with difficulties in various social aspects, including eye contact and facial information processing [28], joint attention [7], and social information processing [1]. Recent research highlights the overlap between autism and AN, leading to poorer psychological outcomes, social and flexibility problems, and worse treatment response in individuals with both conditions [14, 16, 20, 24, 29, 37].

Recent qualitative studies have shed light on the specific challenges encountered in providing support for individuals with co-occurring AN and autism within clinical settings, particularly due to their social communication difficulties. Kinnaird and colleagues [15] interviewed clinicians to explore their experiences working with individuals who have both AN and autism. The findings highlighted that communication problems made establishing therapeutic rapport more difficult, and clinicians had to adjust their communication styles to accommodate the unique needs of each patient. Similarly, another study reviewed case notes on patients with AN and autistic traits, alongside minutes from case study discussions [18]. This study identified communication difficulties as one of the key challenges faced by clinicians when treating individuals with AN and autism, with severity ranging from mild difficulties in articulating thoughts to selective mutism. Overall, the social communicative and emotional profile of autistic individuals may hinder traditional therapeutic approaches that heavily rely on verbal articulation of thoughts and emotions. Hence, there is a need for resources to further understand and support the communication difficulties of autistic individuals in ED treatments.

Implemented at the South London and Maudsley (SLaM) Inpatient ED service in 2018, PEACE (Pathway for Eating disorders and Autism developed from Clinical Experience; peacepathway.org) is a treatment framework designed to address the unique challenges faced by autistic individuals with ED, and is the first systemic approach to tailor standard ED treatment for autistic individuals [33]. As part of the PEACE pathway implementation, comprehensive training was provided for clinicians at the ED service on autism awareness and personalized therapy adaptations [27]. Additionally, resources and workshops were developed to support patients’ sensory sensitivities [17, 31] and communication challenges. As part of the PEACE pathway implementation, a communication passport was introduced, in line with recommendations from the National Autistic Society (NAS) advocating for the use of health passports to facilitate communication between autistic individuals and healthcare professionals [23]. This communication passport was developed based on the NAS’s “My Health Passport” resource [22], originally designed to assist autistic individuals in communicating their needs to healthcare professionals. It was adapted to better suit the setting of an ED service: Questions were condensed to one page in order to streamline the incorporation of the passport as part of the admission process, and questions related to pain and needle injections were replaced with general prompts about dislikes and struggles, and what the care team could do to help. Previous studies have discussed the merits of health passports in supporting communication between patients with developmental disabilities and healthcare providers. These passports varied from simple one-page documents designed for individuals with learning disabilities [5] to more comprehensive documents containing medical history, intended for use in emergency departments [13]. However, no study has yet explored the use of communication passport in an ED service context, particularly among patients reporting autistic characteristics.

The aim of the current study was to explore ED patients’ needs through the use of a brief, one-page communication passport at the SLaM National ED Service. Through explorative qualitative analysis of patient responses, the study aimed at elucidating communication preferences, challenges, dislikes, and support needs among individuals with and without autistic traits.

## Methods

### Participants

Adult patients (see Table 1 for patient demographics) diagnosed with an ED within the SLaM National Inpatient ED Service filled out the communication passport as a routine component of the service’s admission process. The diagnoses of ED were established by qualified clinicians upon the patients’ admission to the service. In total, 38 completed communication passports were sampled and analysed until data reached saturation. This study was approved by the South London and Maudsley NHS Foundation Trust clinical governance committee PPF ID 335.

**Table 1:** Baseline demographics and clinical characteristics

Baseline characteristics	
Age, mean (SD)	31.4 (13.55)
Gender, female *n *(%)	37 (97.4)
*Diagnosis, n (%)*	
AN restrictive subtype	32 (84.2)
AN binge-purge subtype	5 (13.2)
ARFID	1 (2.6)
*Autistic traits, n (%)*	
HAT	14 (36.8)
LAT	21 (55.3)
Unspecified (missing data)	3 (7.9)
*Work and social functioning, n (%)*	
Severe impairment (WSAS: 20–40)	29 (76.3)
Moderate impairment (WSAS: 10–19)	2 (5.3)
Subclinical (WSAS: 0–9)	3 (7.9)
Unspecified (missing data)	4 (10.5)

*AN* anorexia nervosa, *ARFID* avoidant restrictive food intake disorder, *HAT* high autistic traits, *LAT* low autistic traits, *WSAS* Work and Social Adjustment Scale

**Table 2:** Summary of participants’ responses on the communication passport

Guiding questions for data analysis	Corresponding prompt(s) on the communication passport	Themes
What communication preferences do patients have?	“How I would like you to communicate with me”	*Preferred mode of communication*
		Discreet and one-to-one
		Non-verbal
		Repeating information
		*Preferred style and tone*
		Gentle, soft voice
		Transparency and honesty
What sensory difficulties do patients have?	“Sensory needs (e.g. my sensitivity to light, sound, touch, texture, taste, or smell and how you can support me)”	Auditory
		Smell
		Tactile
		Taste and texture of food
		Visual
What special interests do patients have?	“My special interests and strengths are”	Cooking and baking
		Entertainment and leisure
		Education and childcare
		Intellectual pursuits
		Music and performing arts
		Nature and animals
		Religion
		Sport
		Visual arts and crafts
What strengths do patients have?	“My special interests and strengths are”	Personality-related
		Skill-related
What messages do patients wish to convey to the care team?	“Other things you should know about me” “Main message that I would like you to know”	Importance of recognising personal and cultural identity
		Importance of personal relationships
		Wanting to recover
What do patients dislike and struggle with?	“My dislikes and things that I struggle with, and how you can support me”	Managing changes and information overload
		Struggle with expressing needs and emotions
		Dislike imposed control and patronisation
		Struggle with self-compassion and low self-esteem
		Struggle with social attention and interaction
What support do patients need?	“What support do I need communicating in group settings” “Sensory needs (e.g. my sensitivity to light, sound, touch, texture, taste, or smell and how you can support me)” “My dislikes and things that I struggle with, and how you can support me” “You can support me by”	Need support for participating in groups
		Need reassurance and external permission
		Need advanced warning about changes
		Need verbal check-ins
		Need to have conversations not related to illness
		Need space and time alone
		Need to be involved in treatment decision-making

### Materials

#### Demographic information

Data regarding participant age, gender, and diagnosis were collected by clinical team members involved in the study.

#### Autism-Spectrum Quotient, short version (AQ-10)

The AQ-10 [2] is a short screening tool designed to assess autistic characteristics through 10 self-report items and has been widely used in the ED literature [35]. A score of 6 or higher indicates the presence of autistic traits and warrants further full assessment of autism. In this study, patients were allocated to high autistic traits (HAT) and low autistic traits (LAT) groups based on their AQ-10 scores.

#### Work and Social Adjustment Scale (WSAS)

The WSAS [21] is a self-report measure used to evaluate the extent to which a person’s symptoms interfere with their work and social activities and has been utilised in clinical samples in the ED literature [12]. The scale assesses functioning across five areas: work, social leisure activities, private leisure activities, home management, and close relationships. The total score is calculated by adding up all of the items. A WSAS score over 20 suggests moderately severe or worse psychopathology. Scores between 10 and 20 are associated with moderate impairment, and scores below 10 are associated with subclinical populations.

#### Communication passport

**Fig. 1:**
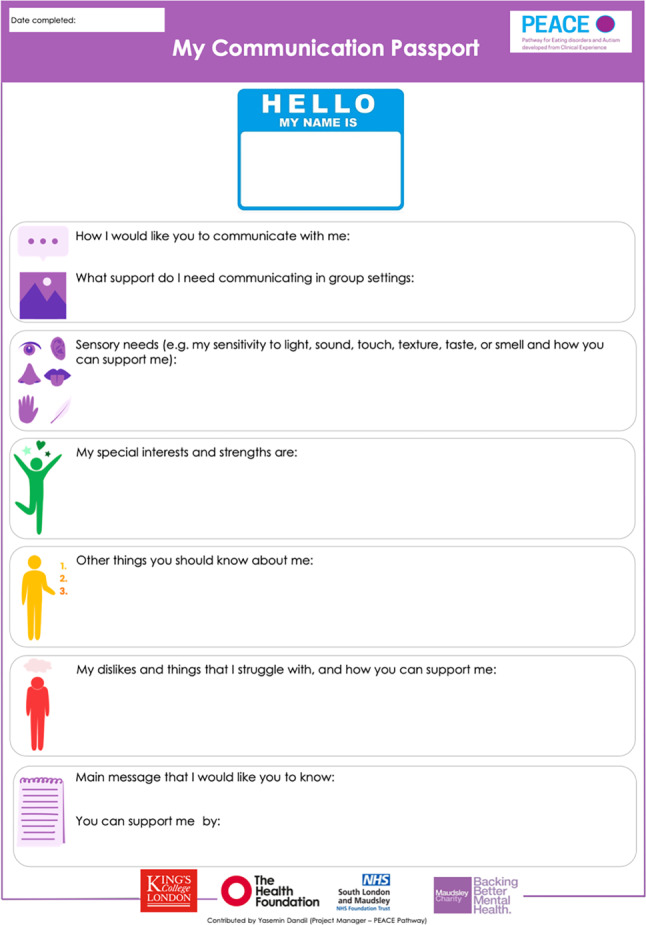
Communication passport, finalised version

The PEACE team piloted the communication passport at SLaM Inpatient ED Service in 2019. It was modified in July 2022 to add one more question (“What support do you need communicating in group settings?”), based on patients’ and clinicians’ feedback. The finalised version of the communication passport starts with a space for the patient’s preferred name and consists of eight questions on a one-page document, all given in a first-person account (Fig. 1):

- How I would like you to communicate with me:
- What support do I need communicating in group settings:
- Sensory needs (e.g. my sensitivity to light, sound, touch, texture, taste, or smell and how you can support me):
- My special interests and strengths are:
- Other things you should know about me:
- My dislikes and things that I struggle with, and how you can support me:
- Main message that I would like you to know:
- You can support me by:

Explanatory coloured images accompany all questions in the communication passport to aid understanding of the questions. The passport is typically completed by the patient with a member of the multidisciplinary clinical team, providing assistance if the patient encounters difficulties in understanding the questions or using the passport. Once completed, a copy of the passport is uploaded to the patient’s electronic clinical record, accessible to the entire care team. Patients and the clinical team are encouraged to use this tool in their everyday routine, particularly when meeting new staff members or attending appointments with professionals they are meeting for the first time. Patients can review their passports during their treatment in case there are changes in their circumstances or preferences. They are also encouraged to use it as a communication tool in other services after they are discharged.

### Analysis

We conducted an explorative qualitative analysis of 38 completed communication passports to investigate patients’ communication preferences, sensory needs, struggles and dislikes, and areas of support required. The data were managed and analysed using the qualitative data analysis software NVivo 14 Lumivero (2023) (NVivo, Version 14, www.lumivero.com). The first author (ZL) read and re-read the data for familiarisation. Seven guiding questions were first proposed based on the original questions on the communication passport to guide the development of the coding framework, including: What communication preferences do patients have?; What sensory difficulties do patients have?; What special interests do patients have?; What strengths do patients have?; What messages do patients wish to convey to the care team?: What do patients dislike and struggle with?; and What support do patients need? Data were coded and organised to generate themes, which were checked by DC against the original data for reliability. Based on the responses collected, we also reflected on how we can further improve the communication passport format.

## Results

Patient demographics and baseline characteristics are outlined in Table 1. The majority of the sample (84.2%) were diagnosed with AN restrictive subtype. Among the participants, 36.8% scored above the cut-off of 6 on the AQ-10 and were classified as HAT patients, with 55.3% classified as LAT patients. The majority of patients (76.3%) displayed severe impairment in work and social functioning, as assessed by the WSAS (scoring above the threshold of 20).

The examination of the entries in the communication passport revealed a range of valuable patient insights, summarised in Table 2. These included their communication preferences, sensory difficulties and needs, special interests and personal strengths, important messages about personal values intended for the care team, struggles and dislikes, and different kinds of support required. We discuss each aspect individually in the following sections, providing a more comprehensive understanding.

### Communication preferences

Patients reported their communication preferences in response to the first question on the communication passport: “How I would like you to communicate with me.” Analysis revealed two predominant themes: patients’ preferred mode of communication, and their preferred tone and style of communication. Regarding their preferred mode of communication, patients highlighted the importance of private, one-on-one conversations away from distractions. When necessary, non-verbal ways of communication such as written materials, visuals, and body language were also helpful. Furthermore, they expressed a need for patience, requesting that information be repeated multiple times to ensure understanding.

Regarding the preferred style and tone of communication, many patients highlighted the importance of kind and gentle communication that is delivered “calmly, kindly, gently” with a “soft voice”. Moreover, patients value transparency and honesty, preferring open and direct conversations regarding their treatment rather than leaving things unsaid or concealed from them. Patients with and without autistic traits gave largely similar responses in this domain. However, only HAT patients mentioned a requirement for information to be reiterated multiple times.

### Sensory sensitivities

Various domains of sensory sensitivities were identified in patients’ response to the prompt “Sensory needs (e.g. my sensitivity to light, sound, touch, texture, taste, or smell and how you can support me)”, including auditory, olfactory, tactile, visual sensitivities, as well as sensitivities to taste and texture of food. Auditory sensitivities were prevalent among the majority of patients, who found noisy environments such as “slamming doors”, “alarms”, “footsteps”, and “loud music on the radio” stressful, although some patients preferred background noise. Following auditory sensitivities, tactile sensitivities were commonly reported, with responses divided into sensory-seeking behaviours, such as the need for stress balls and fidget toys, and sensory-avoidant behaviours, such as discomfort with physical contact like hugging. Additionally, many patients reported sensitivities to bright or flashing lights (“Low light helps me focus. Bright lights are distracting”), strong smells such as perfumes, and preferences regarding taste and texture of food, with some preferring plain and bland foods while others favoured strong tastes (“need flavour and need sweet”). Overall, both HAT and LAT patients reported various sensory sensitivities.

### Special interests and strengths

In response to the prompt “My special interests and strengths are. . .”, patients reported a wide range of interests, which were categorised into: cooking and baking; entertainment and leisure (e.g. favourite TV shows and films, traveling, games, shopping); education and childcare; intellectual pursuits such as reading, science and maths; music and performing arts; nature and animals; religion; sports; and visual arts and crafts. Patients also reported their strengths, categorised into personality-related strengths (such as truthfulness, integrity, willingness to help others, and curiosity) and skill-related strengths (such as communication skills and analytical skills). Both HAT and LAT patients reported a wide range of special interests and strengths with no difference between the two groups.

### Important message to the care team

Patients also used the space for “Other things you should know about me” and “Main message that I would like you to know” to convey significant messages to the care team: firstly, the importance of their personal relationships with family, friends, and pets (“My family and friends all mean a lot to me”); secondly, the significance of their personal identity (“Treat me like a normal person”; “Don’t talk to me like I am an illness”) and cultural identity (including their hometown, preferred pronouns, and autism diagnosis); and thirdly, their determination to improve with treatment (“I want to get better”). Patients with and without autistic traits gave similar responses.

### Dislikes and struggles

In response to “My dislikes and things that I struggle with”, patients reported their dislikes and struggles across five areas: adapting to changes and managing information overload (“[Dislike] schedule that is uncertain”; “[Struggle with] processing information”); expressing needs and emotions (“I struggle to ask for help”); imposed control and patronisation from others (“I don’t like being forced”: “[Dislike] being patronised”); low self-esteem and self-compassion (“[Struggle with] giving myself permission for enjoyable activities”); and navigating social attention and interaction (“Hate being in the spotlight”; “Find it hard to open up to people”). Notably, only HAT patients and none of the LAT patients reported difficulties with changes and information overload.

### Support needed

Several questions on the communication passport prompted patients to outline the kinds of support they require (e.g. “What support do I need communicating in group settings”; “My dislikes and things that I struggle with, and how you can support me”; “You can support me by”). From their responses, seven key support needs were identified. Firstly, patients expressed a need for support with group participation. This involved being invited in during discussions and given the opportunity to contribute and receiving individual check-ins after the group. Patients also reported a need for reassurance and external permission to eat from the care team (“The reassurance that it is okay to eat my meals”). Additionally, they valued receiving advanced warning about any changes to plans (“If a plan is going to change, please tell me”). Furthermore, patients appreciated the care team checking in on their well-being (“Check in with me”; “Asking me what could help in the moment”). They also appreciated conversations on topics unrelated to their illness (“Help by talking about other things outside of [ED]”). Lastly, patients stressed the importance of having personal space and time alone (“I need time on my own”) and expressed a desire to be involved in decisions regarding their treatment and care (“Keep me in the loop”; “Be collaborative with me”). Overall, patients with HAT and LAT reported similar support needs.

## Reflections

This study explored patients’ needs as documented in their communication passports at a specialised inpatient eating disorders service. Overall, the communication passport provided valuable insights into patients’ communication preferences, sensory sensitivities, challenges, and support needed, which would have been otherwise difficult and time-consuming to compile. Furthermore, patients used the passports to share information about their strengths, personal identity, and life beyond the hospital. Through these empowering narratives, the communication passport provides opportunities to discuss personal goals and motivations for recovery during therapy sessions, fostering a deeper understanding between patients and the care team. The insights from the communication passport can also support clinicians’ decision-making in choosing appropriate therapeutic approaches, group activities, and communication strategies tailored to each patient’s needs: for example, adaptations such as providing more frequent sessions and short breaks would allow for more information processing time for patients struggling, and environmental adaptations such as low lighting in the therapy room could benefit patients who report sensitivity to bright light on their passport. This personalised approach has clinical implications for enhancing the overall patient experience and fosters a sense of respect and inclusivity. It also helps to build trust and rapport, leading to better therapeutic relationships.

It is important to note that while patients with both high and low levels of autistic traits reported similar support needs, only those with high autistic traits highlighted difficulties with changes and information overload, expressing a need for information to be repeated multiple times. This is consistent with existing literature, such as the study by Mackie and Fan [19], which suggests that autistic individuals often struggle with information processing due to cognitive and social barriers. This struggle can create communication barriers for autistic individuals within healthcare settings, limiting their access to necessary care [30]. Furthermore, Kinnaird et al. [15] have emphasised the need for healthcare providers to recognise and adapt to the communication styles of autistic patients. The communication preferences identified in this study could serve as key areas for enhancing communication training for healthcare professionals. Additionally, a significant portion of our patient cohort (76.3%), regardless of their autistic status, were assessed using the WSAS to have severe difficulties in social and work areas. This highlights the prevalent social challenges faced by ED patients and the need to improve support in this area.

Overall, providing patients with a communication passport empowers them to actively participate in their care by expressing their communication preferences, needs, and identity. As can be seen from the responses in this study, the passport can serve as a tool for self-expression, enabling patients to assert their rights to accessible and respectful communication. This fosters a sense of agency and autonomy, which is core to recovery from eating disorder [9, 10].

### Limitations

This was a qualitative study and thus represents the experiences of only a minority of people with ED receiving treatment at the service. The majority of patients in our study group had AN, even though we have not excluded other ED subgroups. Therefore, our sample does not represent the full range of ED diagnoses. It should also be noted that the assessment of autistic traits and social functioning in this study was based on self-report questionnaires. These tools do not represent the gold standard diagnostic measures used by clinicians.

### Challenges and improvement

Several logistical challenges emerged during the introduction of the communication passport. Maintaining and updating a communication passport for each patient requires time and resources. In a busy inpatient setting, it may be challenging for the clinical team to consistently refer to and update the passports, leading to potential gaps in communication. Initially, although intended for the entire team, only some members of the care team accessed and utilised the passport. Patients expressed frustration as their specified needs on the passport were occasionally overlooked by team members who had not reviewed it. This issue is echoed by previous studies on health passports. Bell [4] developed a health passport in the style of a traffic light to support patients’ transfer between hospital and community. Despite receiving favourable feedback from hospital staff, this passport was not consistently recognised in the hospital and not routinely reviewed. Similarly, Brodrick et al. [5] reported that the main challenge in the implementation of their health passport was that not all staff were adequately informed about it. In our experience, we found it beneficial to integrate the communication passport into the standard admission procedure and provide education and training about its purpose. To ensure accessibility by the entire team, we began uploading completed passports to patients’ online clinical records and circulating them by email to team members. We also encourage patients to view it as a self-help resource to share with staff members themselves. This empowers them to actively participate in their care by expressing their communication preferences and needs.

To ensure its usefulness, the passport may require regular evaluation and updates. We encourage patients to view the communication passport as a live document that is regularly updated throughout treatment to reflect their evolving needs and preferences. The passport is initially completed on admission and updated prior to discharge to facilitate a smooth transition to other services. Such transitions, such as moving from a ward to supported accommodation, often involve adaptation to new environments and communication challenges. Updating the passport before discharge ensures the accurate transfer of crucial information to the next care provider, promoting continuity of care. However, the practicality of this process needs to be considered. While it offers patients a way to quickly convey their needs and concerns to healthcare professionals, this can increase the challenges for the care team to meet these needs and keep information updated. Therefore, a formal evaluation of how patients use the passport and the perspectives of healthcare professionals is warranted.
